# ASSOCIATION BETWEEN PERCEIVED STRESS IN ADOLESCENCE, BODY WEIGHT AND ROMANTIC RELATIONSHIPS

**DOI:** 10.1590/1984-0462/;2017;35;4;00012

**Published:** 2017-09-21

**Authors:** André de Araújo Pinto, Gaia Salvador Claumann, Pâmella de Medeiros, Rita Maria dos Santos Puga Barbosa, Marcus Vinicius Nahas, Andreia Pelegrini

**Affiliations:** a Universidade do Estado de Santa Catarina, Florianópolis, SC, Brasil.; b Universidade Federal do Amazonas, Manaus, AM, Brasil.; c Universidade Federal de Santa Catarina, Florianópolis, SC, Brasil.

**Keywords:** Adolescence, Psychological stress, Body image, Weight perception, Adolescência, Estresse psicológico, Imagem corporal, Percepção do peso

## Abstract

**Objective:**

To analyze the association between perceived stress in adolescence, body weight and romantic relationships.

**Methods:**

Participants were 2,571 adolescents (56.1% female), with mean age of 16.6±1.2 years, who were students of public schools in Amazonas. The adolescents answered a questionnaire with sociodemographic questions (sex, age group, school year, study shift, maternal schooling and family income) and related to body weight dissatisfaction, romantic relationships (identified by the relationship status - with or without a partner) and perceived stress (dependent variable). Binary Logistic Regression was used to test the association between perceived stress, body weight dissatisfaction and romantic relationships. The analysis was adjusted by sex and age group.

**Results:**

The prevalence of perceived stress was 19.0% (95% confidence interval - 95%CI 17.5-20.3), and was higher among girls (23.2%; 95%CI 21.5-24.5) than boys (13.6%; 95%CI 12.2-14.7). Adolescents with partners (OR 1.76; 95%CI 1.14-2.71) and those who wanted to lose body weight (OR 1.53; 95%IC 1.18-1.98) were more likely to perceive themselves as stressed.

**Conclusions:**

There was an association between perceived stress, relationship status and body weight dissatisfaction. Regardless of sex and age group, the adolescents with a partner and those who wanted to lose weight were more likely to perceive themselves as stressed. Girls should receive special attention, as well as adolescents with partners and those who want to lose body weight.

## RESUMO

**Objetivo:**

Analisar a associação entre estresse percebido na adolescência, peso corporal e relacionamentos amorosos.

**Métodos:**

Participaram do estudo 2.517 adolescentes (56,1% moças), com média de idade de 16,6±1,2 anos, estudantes de escolas públicas estaduais do Amazonas. Os adolescentes responderam a um questionário composto por questões sociodemográficas (sexo, faixa etária, ano escolar, turno de estudo, escolaridade da mãe e renda familiar) e relacionadas à insatisfação com o peso corporal; ao envolvimento em relacionamentos amorosos (identificado pelo status de relacionamento - com ou sem parceiro); e à percepção de estresse (variável dependente). A associação entre estresse percebido, insatisfação com o peso corporal e relacionamentos amorosos foi testada por meio da Regressão Logística Binária, ajustando-se as análises por sexo e faixa etária.

**Resultados:**

A prevalência de estresse percebido foi de 19,0% (intervalo de confiança de 95% - IC95% 17,5-20,3), sendo maior entre as moças (23,2%; IC95% 21,5-24,5) do que entre os rapazes (13,6%; IC95% 12,2-14,7). Os adolescentes com companheiro (*Odds Ratio* - OR 1,76; IC95% 1,14-2,71) e que desejavam diminuir seu peso corporal (OR 1,53; IC95% 1,18-1,98) tiveram maior chance de se perceberem estressados.

**Conclusões:**

Houve associação entre o estresse percebido, o status de relacionamento e a insatisfação com o peso corporal. Independentemente do sexo e da idade, os adolescentes com companheiro e os que desejavam diminuir o peso apresentaram mais chances de se perceberem estressados. Uma particular atenção em relação ao estresse percebido deve ser dada às moças, aos adolescentes com companheiros e àqueles que desejam reduzir seu peso corporal.

## INTRODUCTION

Adolescence can be considered as a critical phase for the development of stress, especially due to the little experience of young people in dealing with conflicting situations, such as those inherent to interpersonal relationships with relatives, friends and romantic partners, besides the one related to responsibilities and school-related tasks.^1^ Stress is an indicator of complex mental health, considered as a risk factor for the onset of problems of depression in adolescence, which can, in more severe cases, lead to suicide^2^. Therefore, it is important to address the attention of professionals directly involved with the adolescents, researchers and public health institutions.

International studies showed that the perception of psychological stress in the adolescent population tends to be higher among girls,^3^ ^,^ ^4^ ^,^ ^5^ regardless of age.^4^ ^,^ ^5^ In Korea,^3^ the prevalence of perceived stress was observed in 30.5% of female participants, against 24.2% of the male participants. In England,^5^ of the adolescents who perceived themselves with high level of stress, 54.5% were female, whereas 45.5% were male. In Brazil, the studies conducted with adolescents^6^ ^,^ ^7^ ^,^ ^8^ are leading to the same direction as international investigations, with prevalence of 30.1% among girls and only 9.4% among boys in Santa Catarina;^6^ In Rio Grande do Sul,^7^ among the adolescents who perceived themselves as stressed, 61.5% were female, and 38.5% were male.

Some situations may explain the higher prevalence of perceived stress among girls, in comparison to boys. Among the typical stressful events in adolescence are problems related to appearance,^3^ such as body image dissatisfaction,^9^ ^,^ ^10^ ^,^ ^11^ which are experienced more intensively by girls, despite also affecting boys. However, the evidence about this subject is still limited,^11^ and studies conducted with this focus in Brazilian adolescents were not identified in the literature analyzed. The will to change body shape is worrisome, once its precedent is a negative perception of the individual about the dimensions of their body image, such as weight dissatisfaction, which is associated with deleterious behaviors related with weight control, poor dietary habits, and also suicidal thoughts and attempts.^9^ ^,^ ^10^ ^,^ ^12^ Results from previous studies conducted with Australian adolescents^11^ and young adults,^13^ as well as Korean adolescents,^9^ revealed that those who aimed at reducing their body weight perceived themselves as being more stressed in relation to their peers. This relationship is mainly based on the exaggerated effort made by adolescents to maintain or lose weight,^9^ and the difficulty to reach the desired shape and/or body weight make them potentially sensitive and stressed.^10^

Besides the problems related with appearance and body, studies have suggested that the beginning of romantic relationships in adolescence,^14^ ^,^ ^15^ as well as in adulthood,^16^ can be considered as a triggering event for stressful situations. However, since this condition is more inherent to adulthood, few studies have emphasized this factor among adolescents.^17^ Still, the perception of stress related with romantic relationships does not vary only with age, but also according to cultures; developed countries, for example, usually have less clear and more open family rules for romantic relationships, in comparison to developing countries.^18^

Therefore, it is likely that stressful events, such as body weight dissatisfaction and romantic relationships, perceived by adolescents from countries that are more economically developed, like Korea^3^ and England,^5^ are not the same as those perceived by Brazilian adolescents. Also, it is important to know these factors in order to guide the implementation of programs focused on the promotion of mental health and on the reduction of risks related with long term stress. Based on that, the objective of this study was to analyze the association between perceived stress in adolescence, body weight, and romantic relationships.

## METHOD

This is a secondary data analysis based on the macrostudy Lifestyle and Health Indicators of High School Students from Amazonas (*Estilo de vida e indicadores de saúde de escolares do ensino médio do Amazonas*), conducted in 2011. This study is a cross-sectional epidemiological survey conducted with adolescents from the public educational system from five cities - Itacoatiara, Manaus, Parintins, Presidente Figueiredo e São Gabriel da Cachoeira -, Selected intentionally, due to the geographic location of Amazonas: most are riverside communities, and that is a barrier for the development of this kind of study. This study was approved by the Human Research Ethics Committee from Universidade Federal do Amazonas (CAAE nº 0302.0.115.000-11).

The target-population was constituted of adolescents of both sexes, aged between 14 and 19 years. According to the State Secretariat of Education from Amazonas (Seduc), when the study was conducted, there were 88,562 students enrolled in the five selected cities - 4,164 in Itacoatiara, 78,498 in Manaus, 4,863 in Parintins, 249 in Presidente Figueiredo and 768 in São Gabriel da Cachoeira. The sample selection in the city of Manaus was carried out in three stages:

1. Proportionally per educational districts (n=6), in which all schools were considered as eligible;
2. Stratified per state public schools, considering the total number of students (large: 500 students or more; medium-sized: from 201 to 499 students; and small: up to 200 students); and
3. Cluster of classes, school year and shift, in which all students in class, at the time of data collection, were invited to participate in the study.

In the cities of Itacoatiara, Parintins and São Gabriel da Cachoeira, due to the low number of schools, stages “2” and “3” were used. In Presidente Figueiredo, a census was conducted in the only two schools of the city.

For the sample size calculation, the equation of Luiz and Magnanini was used,^19^ considering 95%CI, prevalence of 50% (unknown outcome), sampling error of five percentage points and design effect of 1.5. Ten percent was added to the sample to reduce the occurrence of possible losses. Therefore, the sample size necessary for each city was determined - Itacoatiara (580); Manaus (631); Parintins (587); Presidente Figueiredo (264); and São Gabriel da Cachoeira (423) -, accounting for 2,485 students. This number rose to 3,267 due to the sampling process by class clusters, in which the invitation to take part in the study was extended to all students attending the class at the moment of data collection - Itacoatiara (580); Manaus (1,413); Parintins (575); Presidente Figueiredo (249); and São Gabriel da Cachoeira (450). However, due to the difficulties found in the transportation between cities and the inappropriate filling out of the forms, the final number of adolescents included was 2,885. Of those, 368 were excluded for not being in the age group specified for this study. Therefore, 2,517 adolescents were part of this study.

Data collection took place in the schools, on days and time previously scheduled with the school management and the Physical Education teachers. The contact with students happened in two moments. Initially, the students were informed about the importance of research and the objectives proposed, and received the Informed Assent and Consent Forms. In the second meeting, the questionnaire for data collection was applied by a team of researchers that was previously trained. Adolescents who were willing to participate as volunteers and who turned in the Informed and the Assent Forms signed by a person in charge were included (those aged <18 years), or they signed them themselves (age ≥18 years).

The questionnaire “Behavior of adolescents from Santa Catarina” was used (COMPAC), carried out based on instruments already validated for adolescents.^20^ For the sample characterization, the following sociodemographic information was collected: sex (male, female), age group (14-16, 17-19 years); school year (first, second, third grades); study shift (day, evening); family income (up to two wages, from three to five wages, and six wages or more - based on the current minimum wage at the day of data collection, R$ 545.00); and maternal schooling (up to eight years, eight years or more). The adolescents also answered about their relationship status (with or without a partner) to identify their involvement in romantic relationships. Body weight satisfaction was investigated using the question: “Are you satisfied with your body weight?”. The options were:

- yes;
- no, I’d like to gain weight; and
- no, I’d like to lose weight.

Perceived stress (dependent variable) was assessed based on a single question: “How do you describe the level of stress in your life?”. The possible answers consisted of:

- rarely stressed, living well;
- sometimes stressed, living reasonably well;
- almost always stressed, facing problems often; and
- excessively stressed, with difficulties to face life.^21^

For analytical purposes, categories “a” and “b” were grouped and constituted the category “without stress”. And categories “c” and “d”, the category “with stress”, according to the classification used in previous analyses.^6^ ^,^ ^21^

The distribution of adolescents participating in this study according to the variables collected is presented in Table 1. Most were female (56.1%), aged between 17 and 19 years (51.0%), attending the first year of high school (43.8%), during daytime (81.0%). The adolescents were mostly from families earning up to two minimum wages (current at the time) (64.0%), children of mothers with less than eight schooling years (65.4%), without a partner (95.4%). Most adolescents would like to gain weight (38.1%).

**Table 1:**
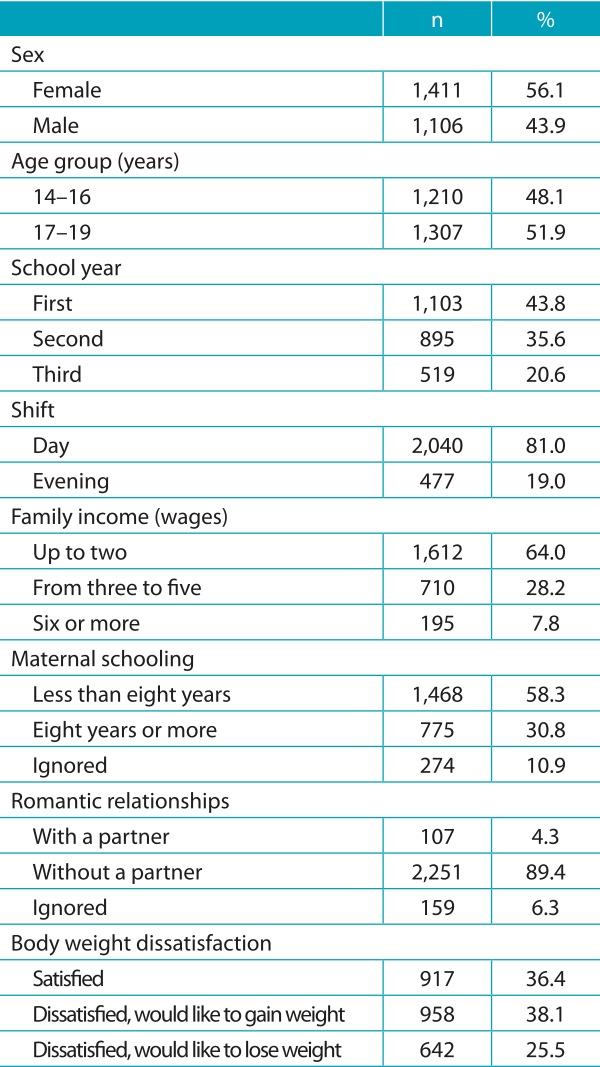
General characteristics of adolescents in the Amazon, 2011.

n: absolute frequency; %: relative frequency.

Descriptive (distribution of absolute and relative frequencies) and inferential analyses were conducted. The chi-square test was used to verify possible differences in the distribution of frequencies of perceived stress among the categories of independent variables. The association between perceived stress, body weight and romantic relationships was tested using the Binary Logistic Regression. There were also the crude and adjusted analyses by sex and age group, since they are considered as confounding variables.^4^ ^,^ ^5^ ^,^ ^6^ ^,^ ^12^ ^,^ ^13^ ^,^ ^21^ The analysis were conducted using the Statistical Package for the Social Sciences (SPSS) software, version 20,0, with 5% significance level.

## RESULTS

**Figure 1:**
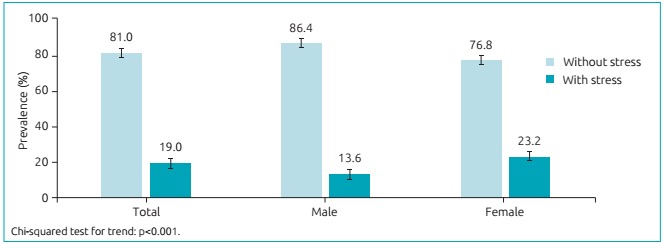
Prevalence of perceived stress in adolescents from, 2011.

The prevalence of perceived stress in adolescents from Amazonas was 19.0% - 95% confidence interval (95%CI 17.5-20.3). By stratifying the sample per sex, it was possible to observe that girls had higher prevalence of stress (23.2%; 95%CI 21.5-24.5), in comparison to boys (13.6%; 95%CI 12.2-14.7) (Figure 1).

Adolescents with a partner and those who would like to lose body weight presented higher prevalence of perceived stress (29.9 and 23.7%, respectively), with significant differences in the distribution of frequencies of these variables, according to perceived stress (Table 2).

**Table 2:**
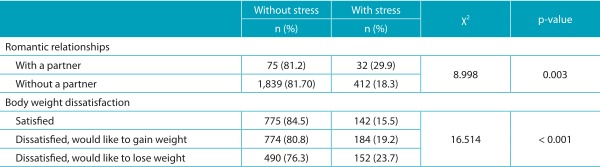
Perception of stress according to body weight dissatisfaction and romantic relationships among adolescents in Amazonas, 2011.

n: absolute frequency; %: relative frequency; χ^2^: chi-square test value.

In the Bivariate Logistic Regression analysis, there was an association between perceived stress and the variables “relationship status” and “body weight dissatisfaction”. After the adjustment by the variables sex and age, it was observed that adolescents with a partner were more likely to (*Odds Ratio* - OR 1.76; 95%CI 1.14-2.71) perceiving themselves to be stressed than their peers without partners. Higher chances of perceived stress (OR 1.53; 95%CI 1.18-1.98) were detected among adolescents who would like to lose body weight, in comparison to those who were satisfied about their weight (Table 3).

**Table 3:**
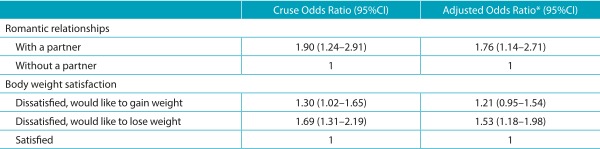
Crude and adjusted association between perceived stress in adolescence, body weight and romantic relationships. Amazonas, 2011.

95%CI: 95% confidence interval. ^*^Adjusted by the variables sex and age group.

## DISCUSSION

This study verified higher prevalence of stress among girls, similarly to the observation in studies with adolescents from Korea (30.5%),^3^ Sweden (47.0%),^4^ and England (54.5%).^5^ In Brazil, studies with adolescents from Santa Catarina (30.1%),^6^ Rio Grande do Sul (61.5%)^7^ and the Federal District (60.0%)^8^ also found similar results. The discrepancy between prevalence rates may be related with the different methodological aspects used in the studies, highlighting sample size, the context in which adolescents are inserted, besides the different instruments used to assess stress.

However, female adolescents are believed to experience stressful events in a more intense manner, and more often than boys, such as helping with household chores, and even helping to care for younger siblings, which possible facilitates that perception.^22^ In addition, girls tend to be more concerned about their appearance and body weight, and adopt fewer attitudes to solve problems regarding physical shape than boys, which, with time, may lead to the development of stress caused by body image dissatisfaction.^23^ As for boys, when confronted by stressful situations, they tend to look for distractions and to reduce the severity of some conflicts more often than girls, looking for more incisive ways to solve unpredicted problems.^24^

Adolescents with partners were more prone to stress. It is worth to mention that the reports available in the literature about the association between the level of stress and relationship status in the adolescent population are still incipient. This in fact becomes a challenge for researchers who try to understand better the association between these factors, since it makes the discussion in the field of stress in this population more difficult, thus contributing with speculation. In spite of that, it is likely that, in adolescence, especially after the age of 14, factors such as the search for family independence and the beginning of romantic relationships are potential stressful events.^15^ ^,^ ^16^ In the social field, the stable union has been pointed out as one of the most favorable events for the development of stress, due to the energy it takes to deal with some conflicting situations, requiring from the other person the need to adjust at times of psychological lack of structure.^25^

Still, in adolescence, it is particularly important that romantic relationships be surrounded by dialogue and the sharing of ideas, in terms of accepting the differences between the partners.^18^ These circumstances, if not absorbed by adolescents, certainly can favor the beginning of fights and discussions, and consequently, the perception of stress, since there is no acceptance of the partner’s space. It is important to point out that the state of Amazonas is very heterogeneous in terms of housing, so the population is comprised of people from different states. Therefore, if family rules are too divergent, the absence of maturity, which is a characteristic of adolescence, may lead to conflicts in relationships, and adolescents can feel more stressed. However, this speculation needs to be tested in other studies.

Among the adolescents from this study, those who would like to lose body weight presented higher chances of perceiving stress. This result is in accordance with the findings in a study about stress and body weight dissatisfaction conducted with adolescents^11^, and in another one conducted with young adults,^13^ which suggest that both aspects of body image, when internalized inappropriately, are risk factors for perceived stress. This association is possibly related with the probable situations of embarrassment or discrimination, which people who do not have and/or see themselves as not having the ideal weight experience in their daily lives.^26^ These circumstances can stimulate the development of different psychological problems, such as stress, once these people may not perceive their body shape as being in accordance with the patterns of a specific society.^13^ This result is particularly important, since some studies have found an association that is directly proportional between body weight dissatisfaction and symptoms of anxiety,^27^ ^,^ ^28^ which can also favor an uncontrolled dietary behavior^29^ and suicidal thoughts.^9^

The results of this study need to be interpreted carefully, considering some limitations. One of them is the cross-sectional design, which does not allow the establishment of cause and effect among the studied variables; the use of a single question to assess stress may have suffered with memory bias, which means adolescents may not have remembered the moments of stress precisely; these data can only be extrapolated for adolescents in schools of the region, except for private ones, and for adolescents who, for some reason, do not attend school.

One of the strong points of the study is the use of a random sample, representative of the adolescents; besides, it is one of the first representative studies in Amazonas. Data collection did not coincide with the period of tests, which may have influenced the prevalence of stress observed in the study. Finally, the results found can be a base for comparison with other studies, besides drawing the attention of the stress issue among adolescents, which should be observed by other professionals.

Based on the results of this study, the authors conclude that, in the population analyzed, there is an association between perceived stress and the variables “relationship status”, and “body weight dissatisfaction”. After the adjustment by the variables sex and age, it was observed that adolescents with partners had more chances of perceiving themselves as being more stressed than their peers without partners. There was a higher prevalence of stress among girls, in comparison to boys. Having a partner and being dissatisfied with body weight, wishing to lose weight, were factors associated with the perception of stress among adolescents from the Amazon.

Since this population is in school, it would be extremely important that educational public policies could consider the possibility of inserting psychologists and nutritionists in the school context, in the sense of working directly with problems related with stress associated with romantic relationships and body weight dissatisfaction. If it is not possible to respond to this suggestion, we reinforce the need for teachers of Physical Education, in their classes focusing on health education, to approach the theme using videos, seminars and scientific lectures addressed to the importance of exercise to maintain body weight and to reduce the levels of stress.
